# Anti-Carbamylated Protein Antibodies as a Reproducible Independent Type of Rheumatoid Arthritis Autoantibodies

**DOI:** 10.1371/journal.pone.0161141

**Published:** 2016-08-18

**Authors:** Ariana Montes, Cristina Regueiro, Eva Perez-Pampin, Maria Dolores Boveda, Juan J. Gomez-Reino, Antonio Gonzalez

**Affiliations:** 1 Laboratorio Investigacion 10 and Rheumatology Unit, Instituto de Investigacion Sanitaria-Hospital Clinico Universitario de Santiago, Santiago de Compostela, Spain; 2 Unit of Diagnosis and Treatment of Congenital Metabolic Diseases, Department of Pediatrics, Instituto de Investigacion Sanitaria-Hospital Clinico Universitario de Santiago, Santiago de Compostela, Spain; 3 Department of Medicine, University of Santiago de Compostela, Santiago de Compostela, Spain; JAPAN

## Abstract

A large fraction of the patients with rheumatoid arthritis (RA) develop specific autoantibodies, which until recently were only of two types, rheumatoid factor (RF) and anti-citrullinated protein antibodies (ACPA). We aimed to replicate important findings about a recently described third type of specific autoantibodies, anti-carbamylated protein (anti-CarP) antibodies, because they have been described based only in the homemade ELISA from a single laboratory. Our study included 520 patients with established RA and 278 healthy controls of Spanish ancestry and it was done with an independently performed ELISA. The prevalence and pattern of environmental, clinical and genetic associations of the anti-CarP antibodies were similar to the previously described. Notably, the presence and titers of anti-CarP correlated with the presence and titers of ACPA, but the anti-CarP antibodies did not share the known genetic and exposure risk factors of the ACPA. In addition, anti-CarP antibodies were independently associated with a higher (10.5%) prevalence of bone erosions. The reproducibility of these characteristics across laboratories and European subpopulations, indicates the wide validity of the results and suggests that determination of anti-CarP antibodies could contribute to explain RA pathogenesis and identify clinically relevant patient subgroups.

## Introduction

Rheumatoid arthritis (RA) is a chronic autoimmune disease characterized by inflammation in multiple peripheral joints with a symmetric distribution, which untreated will lead to bone erosions, deformities, significant handicap and life shortening [1]. RA shows a notable inter-individual variability that poses many problems for treatment and for prediction of evolution [2,3]. RA etiology is complex with an environmental component and a polygenic susceptibility component together with other factors like the larger prevalence in women than in men. The identification of autoantibodies specific of RA has been critical in advancing the understanding of the disease and in the classification of patients with a more uniform clinical pattern. The two well-known autoantibodies are rheumatoid factor (RF), against the Fc of the immunoglobulins, and the anti-citrullinated protein antibodies (ACPA) that are assayed with the anti-cyclic citrullinated peptide (CCP) test. Recently, a third type of specific autoantibodies has been described, which show reactivity against carbamylated proteins (anti-CarP) [4]. They could be useful for prediction, diagnosis or follow-up of the patients [4–9].

A possible limitation of this perspective of progress is that the natural autoantigens recognized by anti-CarP antibodies are unknown, and the assays have been performed with *in vitro* carbamylated proteins from either fetal calf serum or fibrinogen [4]. Differences in preparation of the antigen could lead to conflicting results as has already been observed. In effect, no cross reactivity between anti-CarP or anti-carbamylated fibrinogen antibodies and ACPA has been reported with ELISA performed by the group behind most research in these antibodies, based in the Rheumatology Laboratory of the Leiden University Medical Center [4]. In contrast, extensive cross-reactivity between carbamylated fibrinogen and citrullinated fibrinogen has been reported by other group [10]. Therefore, it is unclear if the characteristics described with the Leiden ELISA for the anti-CarP antibodies are reproducible in different settings. These characteristics show that the anti-CarP antibodies are independent of the risk factors associated with ACPA positive RA [4,9]. Whereas ACPA positive RA is associated with the shared epitope (SE) alleles in the HLA-DRB1 locus, the R620W risk allele of PTPN22 and with smoking [11–13], no association has been observed for the anti-CarP antibodies with any of these genetic or environmental risk factors [9]. On the contrary, the anti-CarP antibodies were associated with the HLA-DRB1*03 allele, which is not a risk factor for ACPA positive RA [9]. However, studies by the same research group have shown that the anti-CarP antibodies seem to have a pathogenic role in the RA disease process. The pieces of evidence pointing in this direction include association with radiological damage of RA, which is independent of the ACPA status [4,7]; detection of anti-CarP antibodies in blood samples taken before the onset of clinical symptoms of RA [5–8]; and experiments in mice showing that immunization with cabamylated peptides was able to elicit erosive arthritis [14].

These results are exciting because they identify a new RA autoantibody type with pathogenic relevance that does not share the known etiological factors with the other autoantibodies. Research in these antibodies will complement our understanding of RA and could be of clinical utility. However, we need independent replication of the findings in different laboratories to be confident on these results. In addition, replication of the characteristics attributed to anti-CarP antibodies in populations with different prevalence of RA risk factors is required to assess their generality.

Here, we present our determination of anti-CarP antibodies in Caucasian Spanish RA patients using antigen and assays prepared in our laboratory. The results confirm the independence of these antibodies from the risk factors that are known for ACPA positive RA and their independent association with RA bone erosions, which are the widely reported characteristics of these antibodies.

## Material and Methods

### Patient and control samples

Sera from 520 RA patients fulfilling 1987 ACR classification criteria [15], and from 278 healthy donors were included in the study. Clinical characteristics of patients (Table 1) have been described elsewhere [16,17]. All patients and controls were Spanish Caucasians, and they have provided their written informed consent. Collection of samples was approved by the Cómite Etico de Investigación Clinica de Galicia. Available information included *HLA-DRB1* genotypes determined by sequencing, *PTPN22 rs2476601* genotypes, anti-CCP2 and IgM RF titers, smoking status (coded as never or ever smoker) and bone erosions as already described [16,17]. SE alleles of *HLA-DRB1* were considered to include the 22 alleles coding for the QKRAA, QRRAA and RRRAA 70–74 amino acids of HLA-DRB1 [18]. A single rheumatologist assessed the most recent hands and feet radiographs of the patients to distinguish between presence/absence of definite bone erosions. Time since disease onset to the set of radiographs was long and very variable (median of 18.0 years (7.9–30.4 IQR) in 20 patients taken at random). This radiographic evaluation was done with total independence of this study.

**Table 1:** Characteristics of the patients with RA included in the study.

Feature	n (%)
Gender (women)	399 (76.7)
Age of disease onset, median (IQR) years	48.6 (35.8–57.2)
Duration of follow-up, median (IQR) years	12.1 (5.3–20.7)
Ever smokers	97 (20.3)
Erosions	325 (65.7)
anti-CCP positive	334 (64.2)
RF positive	301 (60.2)
Shared epitope, carrier	299 (57.5)
*PTPN22*, carrier	137 (26.5)

### Carbamylation and Anti-CarP antibodies detection

*In vitro* carbamylation and ELISA detection of antibodies were done following the described protocol with minor changes [4]. It involved incubation of FCS (F-7524, Sigma-Aldrich, St. Louis, Missouri) at 4 mg/ml with 1M of KCNO, or with 1M KCl as control, during 15 h at 37°C. After incubation, the samples were dialysed against H_2_O with 0.25 NaCl at 4°C during 24h and used as antigen for ELISA. An aliquot was used to assess the efficiency of carbamylation by chromatographic analysis of the hydrolysed amino acids. Acid hydrolysis was done with HCl 6N during 18h at 110°C. A *Biochrom 30* amino acid analyzer (Biochrom, UK) with an ionic exchange column and lithium citrate buffers of different pH were used for separation. Quantification involved post-column reaction with ninhydrin using L-Norleucine as internal standard. The change of lysine to homocitrulline was determined as the fraction of the total amount of amino acids. The anti-Carp FCS antibodies were assayed with ELISA. Separate Nunc MaxiSorp flat-bottom 96 well plates were coated with carbamylated and native FCS overnight at 10 μg/mL in 50 μL of carbonate-bicarbonate buffer 0.1M pH 9.6. The plates were washed with PBS-0.05% Tween and blocked for 6 hours at 4°C with 100 μl of PBS-1% BSA. Diluted serum (50 μL at 1:50 in PBS-1% BSA-0.05%Tween) was incubated on ice overnight. IgG antibodies were detected using ALP-conjugated goat anti-human IgG (Jackson Immunoresearch Europe, UK) and SIGMAFAST p-Nitrophenyl phosphate as substrate following manufacturers' recommendations. Reactivity to native FCS was subtracted from the reactivity to carbamylated FCS and relative quantification was done against serial dilutions of a pool of positive patient sera. The cut-off for positivity was set as the 98^th^ percentile of the healthy controls.

### Statistical analysis

Concordance between positivity of the different antibodies was analyzed with the Goodman and Kruskal's gamma coefficient (γ). Interpretation of his coefficient in a 2x2 contingency table goes from -1.0 = all results are discordant, to +1.0 = all results are concordant. Relationship between antibodies titers was analyzed with Spearman rank correlation (r_s_). Multivariate logistic regression analysis was used to evaluate the effect of each antibody type conditional on the other antibodies. These analyses were used to explore association with *HLA-DRB1*, *PTPN22*, smoking and erosions. Gender, age of diagnosis and time of follow-up were included as covariates without interaction terms. In addition, stratified analyses with contingency tables were done. P values lower that 0.05 were considered statistically significant. All analyses were done with Statistica 7.0 (StatSoft, Tulsa, OK), the eulerAP software was used to draw area proportional Venn diagrams [19].

## Results

Patients showed established RA with clinical characteristics (Table 1) that have already been described [17]. The frequency of RA risk factors in these patients is very different from the reported in Northern Europeans, as already shown [16,17]. Specifically, carriers of SE (53.9%) and smokers (20.3%) were less frequent among our RA patients than in the Dutch (SE = 64.3%, P = 2.3 x10^-4^; smoking = 52.1%, P = 9.7 x10^-29^) and Swedish (SE = 73.6%, P = 3.9 x10^-18^; smoking = 64.9%, P = 1.5 x10^-67^) patients included in previous analysis of the characteristics of the anti-CarP antibodies [4,9].

**Fig 1:**
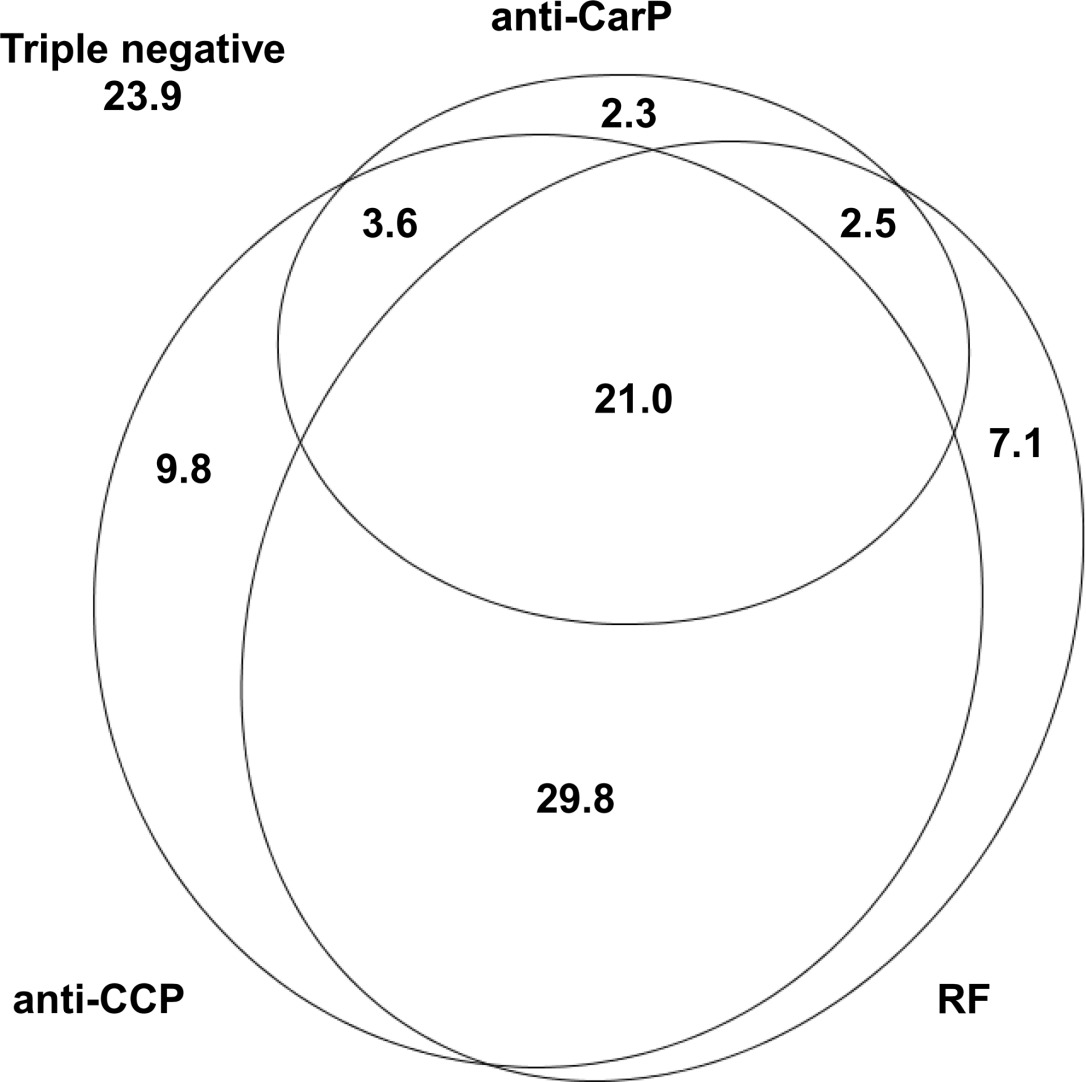
Distribution of patients with RA positive for the different autoantibodies. We observed that anti-CarP antibodies were not associated with the SE alleles (Fig 2A). This lack of association was found in all analyses performed, which included either all the SE alleles together (OR = 1.1, P = 0.6), or only the HLA-DRB1*01 alleles (OR = 0.9, P = 0.7), or the *04 alleles (OR = 1.0, P = 0.9), or the *1001 allele (OR = 1.2, P = 0.7). In addition, we did not observe association in analyses conditional on the anti-CCP status (OR = 0.8, P = 0.4), the RF status (OR = 0.9, P = 0.6) or both (OR = 0.8, P = 0.3). In a similar way, there was no association of the anti-CarP status with the *PTPN22* R620W polymorphism (Fig 2B). Again, this result was consistent in the direct analysis (OR = 0.9, P = 0.7), and in the conditional on other autoantibodies (OR = 0.8, P = 0.5 conditional on anti-CCP; OR = 1.0, P = 0.8 conditional on RF; and OR = 0.9, P = 0.7 conditional in the two other autoantibodies). Finally, we did not observe association of the anti-CarP antibodies with smoking (OR = 1.0, P = 0.9; Fig 2C). We should note that the anti-CCP positive status was associated with the SE alleles (P = 2.4 x 10^−7^), showed a stronger association than the anti-CCP negative status with the *PTPN22* R620W polymorphism (P = 0.002 for anti-CCP positive and P = 0.13 for anti-CCP negative patients), but it was not associated with smoking (P = 0.65) in our patients, as we already have noted previously [16,17]. All these analyses were adjusted for gender, age at disease onset and time of follow-up.

The procedure of *in vitro* carbamylation induced a notable change in the relative abundances of lysine and homocitrulline among the FCS proteins, with a marked decrease in lysine (from 7.8% to 0.2%) and increase in homocitrulline (from 0.7% to 5.1%) (S1 Fig). ELISA using carbamylated FCS led to the identification of 29.4% of the RA patients showing this specificity (Fig 1), which is markedly less than the fraction of anti-CCP positive patients. Accordingly, there was a similar fraction of patients that showed anti-CCP antibodies and lacked anti-CarP antibodies (39.6%), and the status of anti-CarP positivity was moderately concordant with the anti-CCP status (γ = 0.60, 95% CI: 0.47–0.75). A similar degree of concordance was observed with the RF status (γ = 0.56, 95% CI: 0.41–0.72). This level of concordance was clearly lower than the present between anti-CCP and RF (γ = 0.82, 95% CI: 0.75–0.89). In addition, the titers of anti-CarP antibodies (ranging from 120 to over 1511 AU) were correlated with the anti-CCP titers (range from 6 to 6522 units; r_s_ = 0.27, P = 5.3 x 10^−10^). A subset of patients of special interest included the 4.8% patients that were anti-CarP^+^ and anti-CCP^-^. These results are similar to the already reported in other studies [4,7,9], where anti-CarP^+^ patients were 35.6 to 45% and the anti-CarP^+^/anti-CCP^-^ were 4 to 8% of the patients. Given that about half of these patients were RF positive (Fig 1), the fraction of seronegative RA patients was only slightly reduced by the consideration of the anti-CarP^+^ patients from 26.2% to 23.9%.

**Fig 2:**
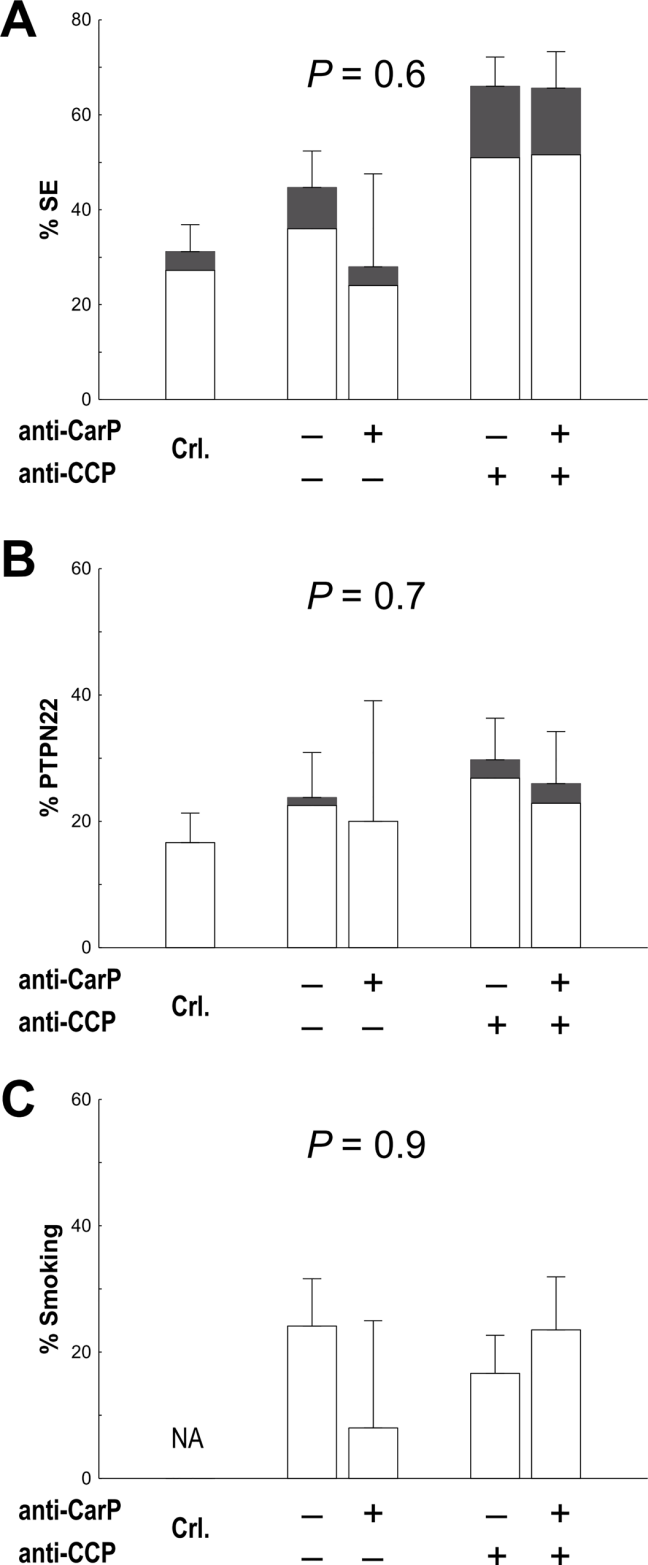
Stratified analysis of RA risk factors according to the anti-CarP and anti-CCP status. Frequency of the A) *HLA-DRB1* SE and B) *PTPN22* R620W genotypes in the different strata. White fraction = heterozygotes, grey fraction = homozygotes. C) Frequency of ever smokers. Error bars represent 95% CI. Crl. = controls, NA = not available. P values were obtained with logistic regression analysis adjusted for gender, age of diagnosis and time of follow-up.

A previous study showed a weak association of HLA-DRB1*03 with anti-CarP antibodies in anti-CCP^-^/anti-CarP^+^ patients (as well as, in anti-CCP^-^/anti-CarP^-^ patients) compared with healthy controls [9]. We did not observe association with the *03 alleles in this or any other subgroup of patients, but there was a nominal deviation in the same direction previously reported (OR = 1.5, P = 0.2 in all the subjects and OR = 1.6, P = 0.2 in the non-carriers of SE alleles).

**Fig 3:**
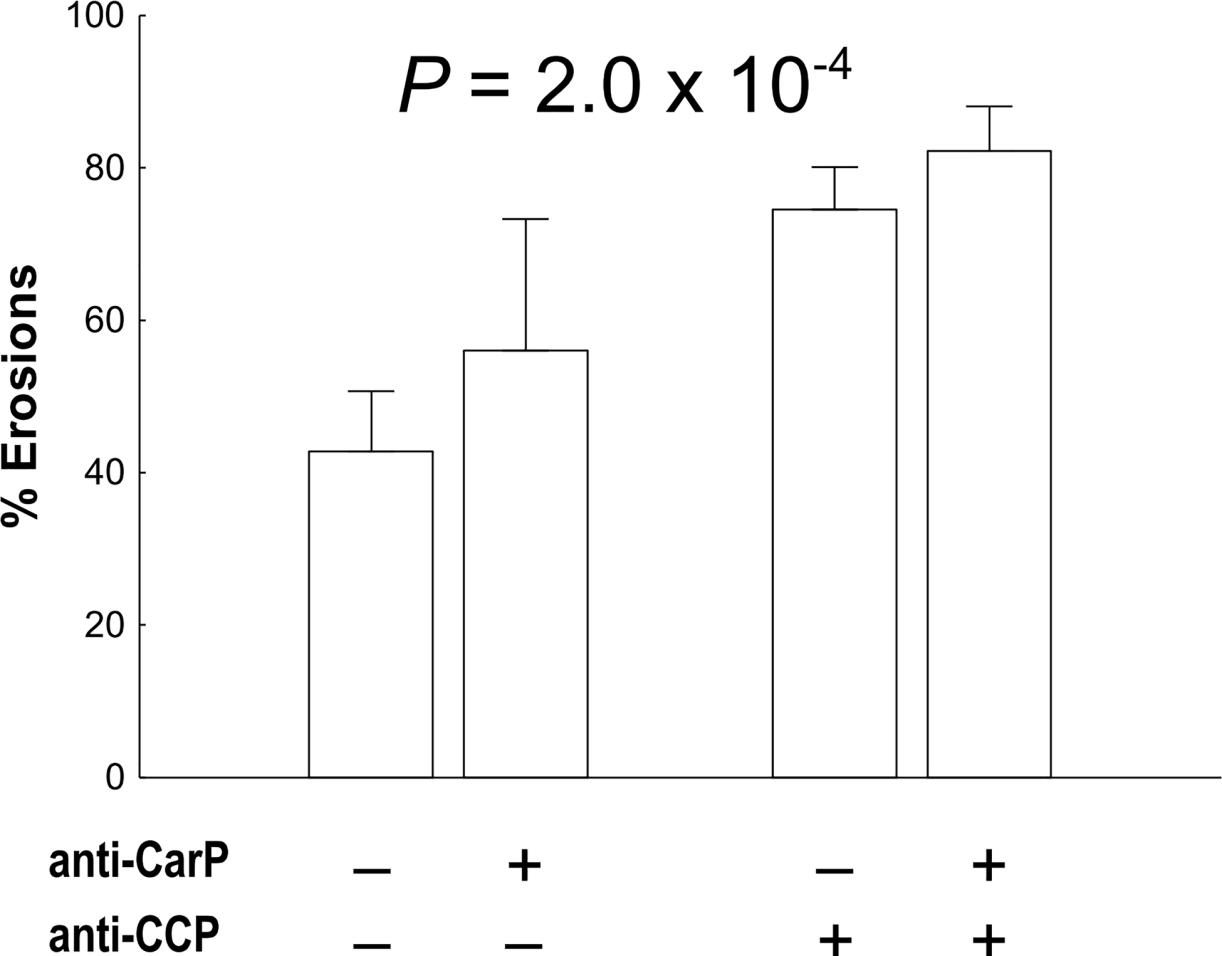
Prevalence of RA bone erosions in anti-CCP and anti-CarP defined subgroups. Error bars represent 95% CI. P value from logistic regression analysis adjusted for gender, age of diagnosis and time of follow-up.

Radiological damage has been associated with anti-CarP antibodies, either as damage at particular time points or as radiological progression [4,7]. The information available from our patients did not allow us studying the same time points or progression; only the presence of bone erosions at the most recent radiographic exam. Presence of erosions was associated with the anti-CarP status (OR = 2.4, P = 2.0 x 10^−4^). A conditional analysis on the presence of anti-CCP antibodies showed also significant association of the anti-CarP status with erosions (OR = 1.76, P = 0.026) indicating the independent contribution of the anti-CarP status. This relationship was also observed with stratified analysis (Fig 3), where the strata including anti-CarP^+^ patients showed numerically more erosions (a mean of 10.5%) than the anti-CarP^-^ patients.

## Discussion

We have replicated the following characteristics of anti-CarP antibodies: they were specific of RA, but less sensitive than the anti-CCP antibodies and less correlated with them than RF; in addition, the anti-CarP antibodies were not associated with the RA risk factors, the *HLA-DRB1* and *PTPN22* risk alleles or with smoking; on the contrary, they were associated with the presence of erosions with independence of the ACPA status. These characteristics have previously been described in studies done with an ELISA we have replicated in a different laboratory and in European subpopulations with notably different prevalence of RA risk factors [4,7,9]. Our results provide in this way wide validity to the anti-CarP antibody characteristics that define them as a second system of autoantibodies targeting post-transcriptional modifications of endogenous proteins in the pathogenesis of RA.

The characteristics of anti-CarP antibodies replicated here provide new opportunities for the understanding of RA pathogenesis and for classification of patients. They could facilitate the discovery of new risk factors specific of the subset of patients producing the anti-CarP antibodies. In this regard, the antecedent of ACPA is very illustrative, as their discovery has led to the identification of the best-known pathogenic pathway in RA [1,20–22]. This pathway includes smoking and periodontal disease as sources of citrullination of proteins, specific *HLA-DRB1* alleles to present citrullinated peptides, and other genetic factors as positive modulators of antibody mediated autoreactivity; epitope spreading together with maturation of the antibody response as steps preceding clinical onset of RA, and the effect of ACPA in stimulating osteoclasts for the RA bone erosions. For anti-CarP, it would be necessary to identify specific risk factors, to analyze if they share with ACPA mechanisms to escape immunological tolerance as targeting neoantigens, as well as, epitope spreading and maturation. It would be also necessary, to clarify the mechanisms leading to the increased risk of radiological damage associated with the anti-CarP antibodies. Are they triggering osteoclast differentiation and activation as the anti-citrullinated vimentin antibodies [23]? Are they cooperating with other antibodies to erosions, as indicated for ACPA and RF [24]?

In addition, our results seem to anticipate that anti-CarP antibodies could have significant clinical utility. The presence of these antibodies in a fraction of ACPA negative patients and their association with radiographic progression, as well as their presence preceding the onset of arthritis, have been signaled as indication of their possible utility [4–8]. The two first features were assessed and replicated in our patients. The utility of these changes for individual patients is still unclear and would need to be specifically addressed. Other possible area of utility, not yet addressed, is as biomarkers of response to treatment.

In conclusion, we think it is already possible to consider the anti-CarP antibodies as independent RA autoantibodies that do not share the same risk factors with ACPA, but that are associated with radiological damage. Demonstration of the wide reproducibility of these characteristics makes of them a firm ground on which to base further studies to clarify the anti-CarP role in the clinic and in RA pathogenesis.

## Supporting Information

S1 Fig **Chromatogram showing separation of digested amino acids from A) native FCS, and B) *in vitro* carbamylated FCS**.
(PDF)

S1 File Excel file with the individual subject data included in this manuscript. (XLS)
