# Supplementary material for: Anti-Carbamylated Protein Antibodies as a Reproducible Independent Type of Rheumatoid Arthritis Autoantibodies
Source: PLoS One. 2016 Aug 18;11(8):e0161141. doi: 10.1371/journal.pone.0161141 (PMC4990271; doi:10.1371/journal.pone.0161141)
Supplement: S1 Fig — Chromatogram showing separation of digested amino acids from A) native FCS, and B) in vitro carbamylated FCS. (PDF) [file pone.0161141.s001.pdf]

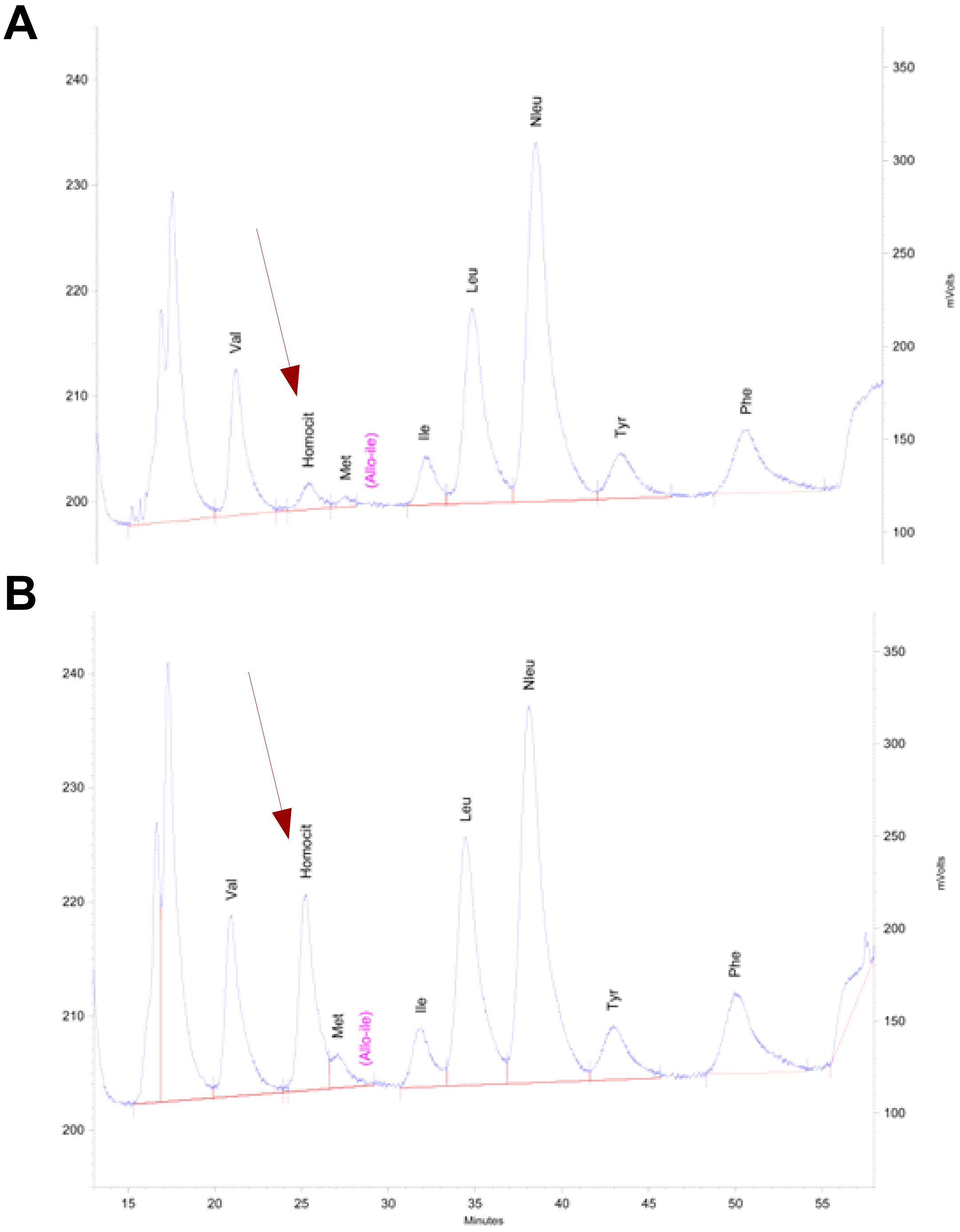


**S1 Figure:** Chromatogram showing separation of digested amino acids from A) native FCS, and B) *in vitro* carbamylated FCS. The two sources of amino acids were used for ELISA as either background or specific target, respectively, in the detection of anti-CarP. Focus was placed in homocitrulline (arrows) and the spiked L-Norleucine standard. Lysine run at later times.
